# Restoring the Patient’s Pre-Arthritic Posterior Slope Is the Correct Target for Maximizing Internal Tibial Rotation When Implanting a PCL Retaining TKA with Calipered Kinematic Alignment

**DOI:** 10.3390/jpm11060516

**Published:** 2021-06-04

**Authors:** Alexander J. Nedopil, Connor Delman, Stephen M. Howell, Maury L. Hull

**Affiliations:** 1 Orthopädische Klinik König-Ludwig-Haus, Lehrstuhl für Orthopädie der Universität Würzburg, 97074 Würzburg, Germany; 2 Department of Biomedical Engineering, University of California, Davis, CA 95616, USA; sebhowell@mac.com (S.M.H.); mlhull@ucdavis.edu (M.L.H.); 3 Department of Orthopedic Surgery, University of California, Davis, CA 95817, USA; cdelman@ucdavis.edu

**Keywords:** total knee replacement, total knee arthroplasty, kinematic alignment, slope, rotation

## Abstract

Introduction: The calipered kinematically-aligned (KA) total knee arthroplasty (TKA) strives to restore the patient’s individual pre-arthritic (i.e., native) posterior tibial slope when retaining the posterior cruciate ligament (PCL). Deviations from the patient’s individual pre-arthritic posterior slope tighten and slacken the PCL in flexion that drives tibial rotation, and such a change might compromise passive internal tibial rotation and coupled patellofemoral kinematics. Methods: Twenty-one patients were treated with a calipered KA TKA and a PCL retaining implant with a medial ball-in-socket and a lateral flat articular insert conformity that mimics the native (i.e., healthy) knee. The slope of the tibial resection was set parallel to the medial joint line by adjusting the plane of an angel wing inserted in the tibial guide. Three trial inserts that matched and deviated 2°> and 2°< from the patient’s pre-arthritic slope were 3D printed with goniometric markings. The goniometer measured the orientation of the tibia (i.e., trial insert) relative to the femoral component. Results: There was no difference between the radiographic preoperative and postoperative tibial slope (0.7 ± 3.2°, NS). From extension to 90° flexion, the mean passive internal tibial rotation with the pre-arthritic slope insert of 19° was greater than the 15° for the 2°> slope (*p* < 0.000), and 15° for the 2°< slope (*p* < 0.000). Discussion: When performing a calipered KA TKA with PCL retention, the correct target for setting the tibial component is the patient’s individual pre-arthritic slope within a tolerance of ±2°, as this target resulted in a 15–19° range of internal tibial rotation that is comparable to the 15–18° range reported for the native knee from extension to 90° flexion.

## 1. Introduction

Total knee arthroplasty (TKA) should restore the native, or healthy, knee’s resting length of the posterior cruciate ligament (PCL) throughout the range of motion to provide stability and to not over or under constrain the knee [1]. A tibial component set in a posterior slope that tightens or slackens the PCL in flexion can decrease the range of motion, increase the risks of tibial component subsidence and polyethylene wear, cause anterior tibial subluxation, and anteroposterior instability which can lead to pain, effusion, and impaired function [1,2,3,4,5,6,7].

The correct target for setting the posterior slope with PCL retention is debatable and depends on the alignment method. A target recommended for mechanical alignment (MA) is 3–7° of the posterior slope [8]. However, a 3–7° range does not account for the 20° inter-individual range of the native posterior slope, and its use changes PCL tension in most knees [8,9,10]. Because the PCL tension in flexion drives tibial rotation, setting the tibial component to an incorrect slope might cause a loss of internal tibial rotation, thereby compromising the coupled reduction in the Q-angle throughout knee flexion, adversely affecting the retinacular ligaments’ tension and patellofemoral tracking [11,12,13,14].

**Figure 1:**
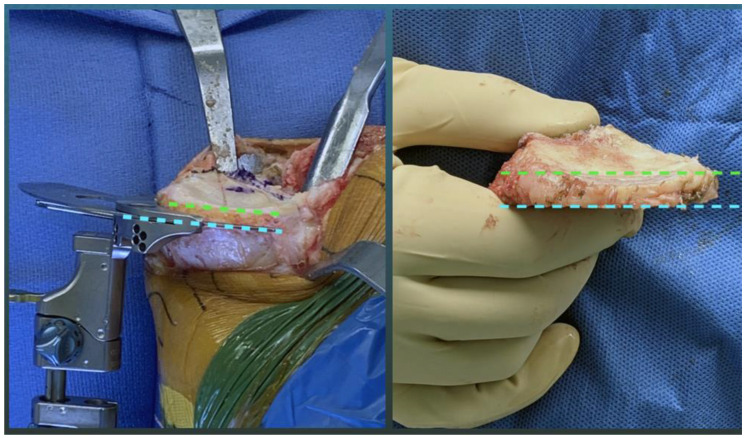
Intraoperative photographs of the medial side of a right knee show the method of setting the tibial resection (blue dotted line) parallel to the medial joint line (green dotted line) by adjusting the plane of an angel wing inserted in the tibial guide (**left**) and the visual verification check showing the tibial resection matches the patient’s native slope (**right**).

In contrast to MA, the recommended slope target for calipered kinematic alignment (KA) is to restore the patient’s individual pre-arthritic posterior slope when retaining the PCL. The caliper technique, which does not release ligaments, sets the femoral and tibial components within 0 ± 0.5 mm of the patient’s individual pre-arthritic distal and posterior femoral joint lines [15,16]. Intraoperatively, the tibial resection slope is set parallel to the medial joint line’s posterior slope by adjusting the plane of an angel wing inserted in the tibial guide (Figure 1). Since the posterior slope on a lateral radiograph is minimally affected by arthrosis, as long as the medial and lateral tibial plateaus closely superimpose, the difference between the pre and postoperative slope can determine the angel wing technique’s accuracy [7,8,9].

**Figure 2:**
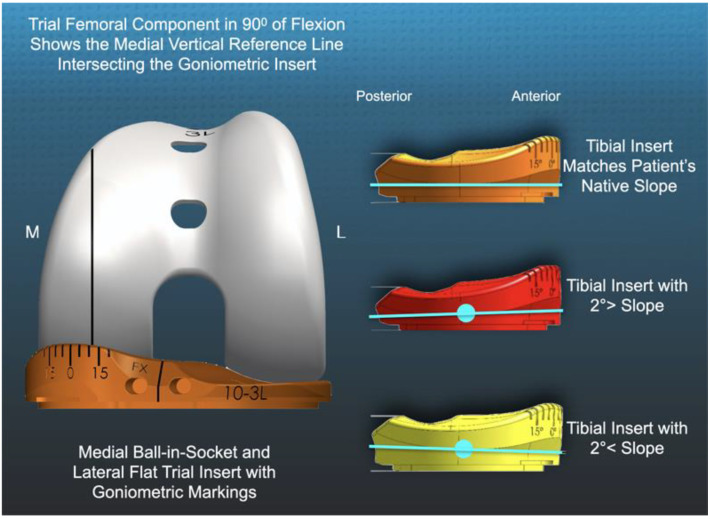
Schematics of a left TKA show the −12° of internal tibial orientation of the trial insert goniometer relative to the medial femoral condyle (left), and the method of creating the tibial inserts with a 2°> (=2° increased) and 2°< (=2° decreased) slope by pivoting the articular surface about the center (blue circle) of the insert that matched the patient’s native slope.

There is a presumption that when a calipered KA retains the PCL and uses components that closely match the surface conformity of the native knee, the coupled internal rotation during passive flexion is also restored. Native knee dissections and image analysis by Freeman and Pinskerova showed that the medial femoral condyle behaves similarly to a ball-in-socket joint, and the lateral tibia and posteriorly mobile lateral meniscus form a flat articular surface, causing the tibia to internally rotate about the center of the medial compartment. The native knee’s 15–18° range of internal tibial rotation from extension to 90° is a desirable arc of motion for TKA [11,17]. A trial insert with native knee conformity and a novel, built-in goniometer can intraoperatively measure the patient’s specific tibial orientation and the degree of internal tibial rotation during flexion (Figure 2). It is unknown whether deviations from the patient’s individual pre-arthritic posterior slope and the corresponding change in PCL tension adversely affect the tibial rotation.

Accordingly, the present study determined in 21 patients, using the insert with the novel built-in goniometer, whether there was a difference between the pre/postoperative posterior slope, and whether trial inserts that matched or deviated ±2° from the patient’s individual pre-arthritic slope changed the patient’s specific tibial orientation in extension and 90° flexion and internal tibial rotation in this range of flexion. The goal of the study was to test the hypotheses that (1) the visual method restores the patient’s individual pre-arthritic slope with good reproducibility, and (2) the patient’s individual pre-arthritic slope is the correct target within a tolerance of ±2° for a calipered KA TKA because it restores a 15–19° range of internal tibial rotation that is comparable to the native knee.

## 2. Materials and Methods

Our institutional review board approved the retrospective study (IRB 1632230-1). Between mid-May 2020 and early June 2020, two surgeons treated 36 consecutive patients with a primary TKA using a calipered KA, PCL retention, and patella resurfacing through a midvastus approach. Each patient fulfilled the Centers for Medicare and Medicaid Services guidelines for medical necessity for the TKA treatment, including: (1) radiographic evidence of Kellgren–Lawrence grade II to IV arthritic change or osteonecrosis; (2) any severity of clinical varus or valgus deformity; (3) and any severity of flexion contracture. Patients were treated with an implant designed by Freeman and Pinskerova, which featured a spherical medial femoral condyle and an insert with a medial ball-in-socket and a lateral flat articular surface (GMK Sphere, Medacta International, Available online: www.medacta.com (accessed on 31 May 2021)) (Figure 2). The implant manufacturer provided 3D printed one-time use trial goniometric inserts in three different slopes (i.e., matching patient’s pre-arthritic slope, 2°> slope, 2°< slope). The three slopes were sterilized and packed for surgical use in 10-, 11- and 12-mm thicknesses and sizes 3, 4, and 5 left and right tibial baseplates (Figure 2) [18,19]. The implant manufacturer provided 3D printed one-time use trial goniometric inserts in three different slopes (i.e., matching patient’s slope, 2°> slope, 2°< slope) in 10, 11, and 12 mm thicknesses for sizes 3, 4, and 5 left and right tibial baseplates (Figure 2). A total of thirty-six consecutive primary calipered KA TKAs were performed to assess 21 knees with the novel insert goniometer because some patients used implant sizes other than those available (i.e., sizes 14 and 17 inserts, and sizes 1, 2, and 6 tibial baseplates), and because some sizes of insert goniometers had been used and were no longer available. The tibial baseplate has an anatomically shaped footprint and a posterior cut-out for retention of the PCL that, when best fit to the tibial resection, sets the internal–external rotation so that the anterior–posterior (AP) axis is parallel to the flexion–extension (FE) plane of the native knee [20]. The first TKAs with an insert thickness and size selected for implantation that matched an available sterile triplet of trial goniometric inserts were studied.

The sample size calculation used the effect of deviating the slope by 2 degrees from the patient’s pre-arthritic slope on passive internal rotation. Assuming a Type I error (alpha) of 0.05, a power (1-beta) of 80%, a minimum difference to detect a 3° change in rotation, and a standard deviation of ±6°, the sample size was 18 patients trialed with three different inserts. Twenty-one patients were included in the study consisting of 67% females with a mean age at the time of surgery of 70 ± 8 years (56 to 81) and a mean BMI of 29 ± 5 kg/m^2^. Descriptive statistics of preoperative clinical characteristics, knee conditions, and function of included (*n* = 21) and not included (*n* = 15) patients are shown (Table 1). Preoperatively, there were no significant differences in age, proportion of women, body mass index, extension, flexion, varus or valgus deformities, Oxford Knee Score, Knee Society Score, or Knee Function Score between included and not included patients, which reduced the risk of a selection bias that could limit the generalization of the study’s findings.

### 2.1. Overview of Unrestricted Calipered KA Technique and Accuracy Analysis of Component Placement

The following is an overview of the previously described unrestricted calipered KA technique performed through a midvastus approach using intraoperatively recorded verification checks and following a decision-tree [21]. For the femoral component, the varus–valgus (VV) and IE orientations and the AP and proximal–distal (PD) positions were set coincident with the patient’s individual pre-arthritic distal and posterior joint lines by adjusting the calipered thicknesses of the distal and posterior femoral resections to within 0 ± 0.5 mm of those of the femoral component condyles after compensating for cartilage wear and the kerf of the saw blade. The basis for setting the distal and posterior femoral resection guide is knowing that the varus and valgus grade II to IV Kellgren–Lawrence osteoarthritic knees have negligible bone wear at 0° and 90°, and that the mean full-thickness cartilage wear approximates 2 mm [22]. An accuracy analysis showed these steps restore the distal lateral femoral joint line of 97% of patients within the normal left to right symmetry and set the IE orientation of the femoral component with a deviation of 0.3° (external) ±1.1° from the KA target of the FE plane of the patient’s knee [15,16,23,24].

The surgeon followed six options in a decision-tree to set the VV and posterior slope orientation of the tibial component to restore the patient’s pre-arthritic tibial joint line and limb alignment and balance the knee by restoring the native tibial compartment forces [23,24,25]. The varus–valgus orientation of the proximal tibial resection was adjusted working in 1°–2° increments until there was negligible medial and lateral lift-off from the femoral component during a varus–valgus laxity assessment with the spacer block and trial tibial insert. An accuracy analysis showed these steps restore the proximal medial tibial joint line of 97% of patients within the normal left to right symmetry [16,24,26]. The method for visually selecting the posterior slope was to set an angel wing, inserted through the tibial guide’s medial slot, parallel to the patient’s pre-arthritic slope (Figure 2). A three-dimensional accuracy analysis in osteoarthritic varus knees reported a 0° mean difference between the patient’s individual pre-arthritic and tibial component’s posterior slope [27]. A best fit of the largest anatomically shaped trial tibial baseplate inside the cortical rim of the proximal tibial resection method set the IE orientation and AP and medial–lateral (ML) positions. An accuracy analysis showed a mean 2° (external) ± 5° deviation of the IE orientation of the tibial component from the KA target of the FE plane of the patient’s knee [15,16,20,27,28,29].

The following steps determined the optimal insert thickness within a ±1 mm target. Place the knee in 90° flexion and palpate the PCL to verify that it is intact. Insert a goniometric tibial insert that matches the thickness of the spacer block. Place the knee in extension and verify that the knee hyperextends a few degrees, such as the pre-arthritic knee. When the knee has a flexion contracture, insert a thinner insert or release the posterior capsule. Verify that the VV laxity is negligible in full extension, the lateral compartment has a 3–4 mm gap and the medial compartment a negligible gap with the knee in 15°–30° flexion. When necessary, fine-tune the VV plane of the tibial resection. Place the knee in 90° flexion and determine whether passive IE rotation of the tibia approximates ±15°, such as the native knee [28].

### 2.2. Method for Radiographically Measuring the Preoperative Tibial Slope and Postoperative Tibial Component Slope

**Figure 3:**
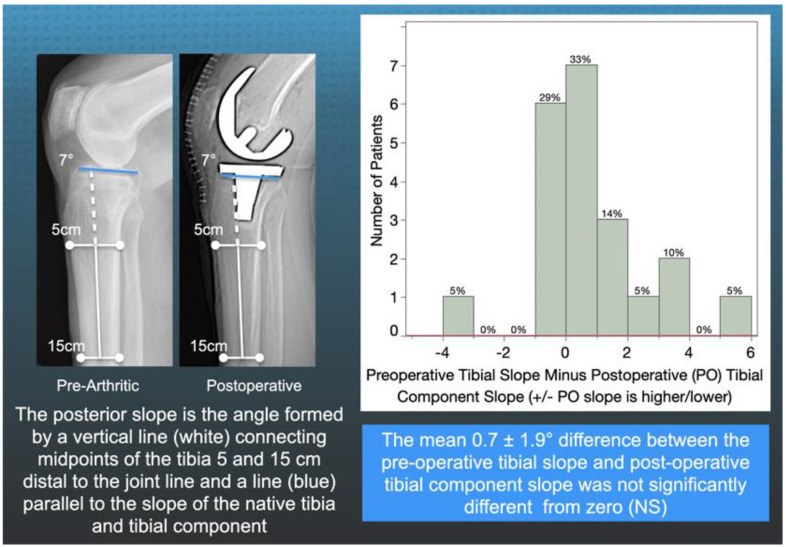
The figure shows the radiographic method for measuring the posterior slope of the preoperative tibia and postoperative tibial component (**left**) and the distribution of the difference in slope for the 21 patients as a measure of the reproducibility in setting the tibial component to the patient’s native slope using the angel wing (**right**).

One author (A.J.N) measured the slope of the patient’s tibia on a preoperative radiograph and the slope of the tibial component on a postoperative computer tomography scanogram using a previously described method with a 0.89 interobserver intraclass coefficient indicative of good interobserver agreement [4,26] (Figure 3).

### 2.3. Method of Measuring the Orientation of the Tibia with Trial Goniometric Insert

**Figure 4:**
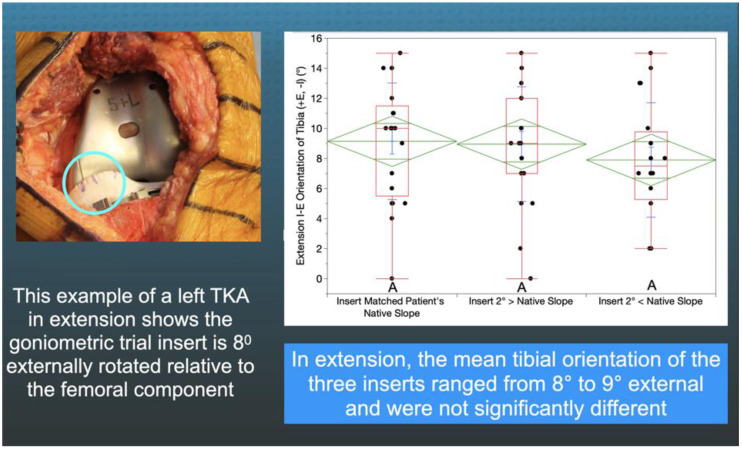
The figure shows an intraoperative photograph of the insert goniometer of a left TKA in extension reading 8° of external tibial orientation (**left**) and box plots of 21 patients that show the mean external tibial orientation was not significantly different between the three inserts (**right**). The top and bottom edges of the green diamond indicate the 95% confidence interval limits.

**Figure 5:**
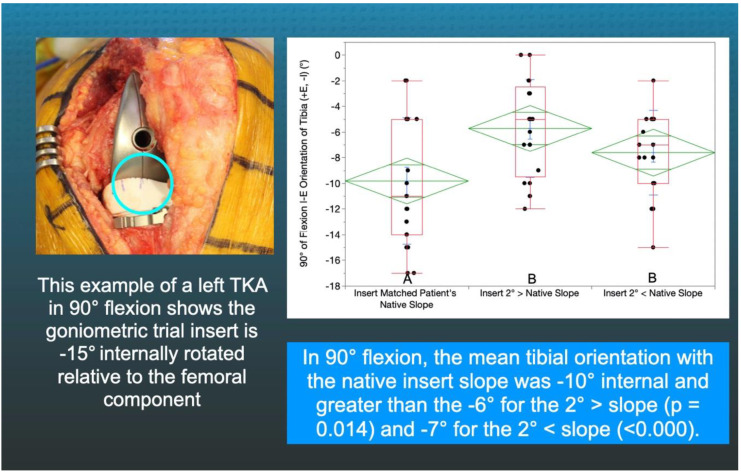
The figure shows an intraoperative photograph of the insert goniometer of a left TKA in 90° flexion reading −15° of internal tibial orientation (**left**) and the box plots show the internal tibial orientation for 21 patients and the insert slopes with different letters are significantly different (**right**).

A goniometric trial insert that matched or deviated 2°> (=2° increased slope) and 2°< (=2° decreased slope) from the patient’s individual pre-arthritic slope was randomly selected and inserted (Figure 2). The surgeon reduced the patella, placed the patient’s heel on the back of the wrist, and lifted the leg to passively extend the knee without applying an IE moment to the ankle. The trial insert goniometer measured the IE tibial orientation relative to the femoral component (+external/−internal) with the knee in extension (Figure 4). The surgeon flexed the knee to 90° and rested the foot on the operating table, and the goniometer measured the tibial orientation (Figure 5). In random order, the surgeon inserted the two remaining inserts and repeated the tibial orientation measurements.

### 2.4. Statistical Analysis

Data were analyzed using statistical software (JMP^®^ Pro 15.2.1, Available online: www.jmp.com (accessed on 31 May 2021), SAS, Cary, NC, USA). The mean and standard deviation described the continuous variables. A Student’s paired t-test determined whether the pre-arthritic and postoperative posterior tibial slope was radiographically different. A mixed-model repeated measured analysis with three fixed effects (i.e., trial goniometer insert that matched and deviated 2°> and 2°< from the patient’s pre-arthritic slope) determined whether there was a difference in mean tibial orientation in extension and at 90° flexion and an internal tibial rotation from extension to 90° between the three insert slopes. For each analysis, a Tukey’s Honest Significant Difference (HSD) post hoc test determined differences between all pairs of insert slopes. Significance was *p* < 0.05.

To quantify reproducibility, two observers (SMH and AJN) measured the slope of the tibia in seven knees. A two-factor mixed-model analysis of variance (ANOVA) with random effects computed the intraclass correlation coefficient (ICC). The first factor was the observer (2 levels), and the second was the patient (7 levels). ICC value of 0.89 indicated good reproducibility for the measurement of tibial slope.

## 3. Results

**Figure 6:**
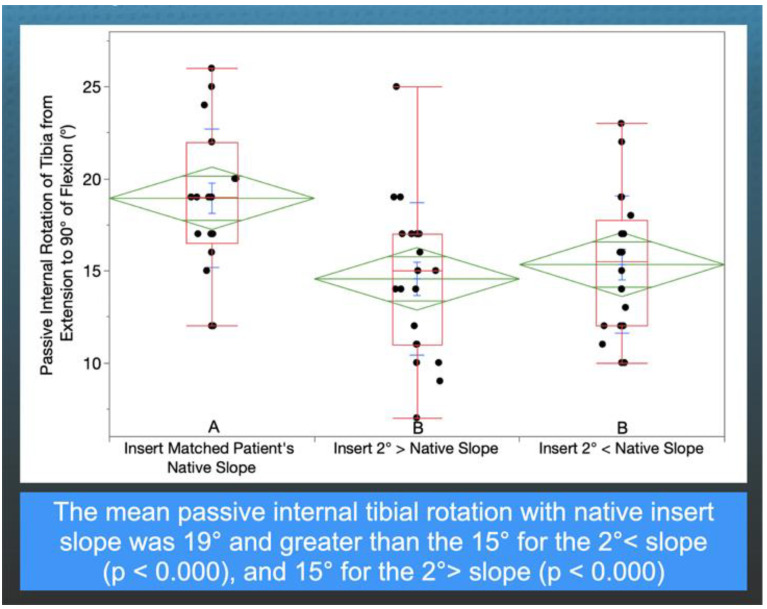
The box plots show the internal tibial rotation from extension to 90° flexion for 21 patients, and the insert slopes with different letters are significantly different.

There was no difference between the radiographic preoperative and postoperative slope (0.7 ± 3.2°, NS), and 75% (16/21) of patients had a <2° difference (Figure 3). In extension, the tibial orientation of the three inserts with different slopes was comparable and ranged from 8° to 9° external (NS) (Figure 4). At 90°, the tibial orientation with the pre-arthritic slope insert was −10° internal and greater than the −6° for the 2°> slope (*p* = 0.014) and −7° for the 2°< slope (<0.000) (Figure 5). From extension to 90° flexion, the passive internal tibial rotation with the pre-arthritic slope insert was 19° and greater than the 15° for the 2°< slope (*p* < 0.000), and 15° for the 2°> slope (*p* < 0.000) (Figure 6).

## 4. Discussion

Knowing the target for setting the posterior slope when performing a calipered KA with PCL retention is necessary to restore internal tibial rotation and the coupled reduction in the Q-angle of the native knee throughout knee flexion and optimize the retinacular ligaments’ tension and patellofemoral tracking. The most important findings of the present study of 21 patients were that (1) the visual method restored the patient’s individual pre-arthritic slope with good reproducibility, and (2) the pre-arthritic slope is the correct target within a tolerance of ±2° for a calipered KA TKA because it restored a 15–19° range of internal tibial rotation that is comparable to the pre-arthritic knee [11,29].

The radiographic reproducibility of the visual method that set the tibial resection plane by aligning an angel wing in the tibial resection guide parallel to the patient’s pre-arthritic joint line was comparable to a three-dimensional shape registration analysis between arthritic surface models segmented from preoperative magnetic resonance imaging scans and resected surface models segmented from postoperative computed tomography scans [27] (Figure 3). Setting the tibial resection to the patient’s pre-arthritic slope is necessary to reduce the risks of tibial component subsidence and posterior polyethylene wear from not restoring the native PCL tension in flexion [4,6]. A study of thirty-three cemented MA TKA with PCL retention using instrumentation designed to cut the tibia with 0° posterior slope reported that ten tibial components had at least 2 mm of subsidence. The subsided tibial components had an 8° mean difference from the patient’s pre-arthritic (i.e., preoperative) posterior slope. The non-subsided tibial components were within ±2° of their individual pre-arthritic slope [6]. A study of cemented calipered KA TKA with PCL retention reported 7 out of 2725 patients with tibial component failure from posterior subsidence or polyethylene wear with a 7° greater slope than the pre-arthritic slope [4]. A varus mechanism was not found to be associated with early tibial component failure after KA, whereas varus overload causing medial bone collapse and varus subsidence of the tibial component is responsible for a comparable—if not higher—incidence of 0.7% revisions after MA. Posterior subsidence generates cement debris, which leads to osteolysis of the tibia and accelerates the subsidence of the baseplate [30]. Hence, cutting the tibia to restore the patient’s pre-arthritic slope is the correct target for reducing the risk of tibial component failure when performing a calipered KA and MA TKA with PCL retention.

The inserts with a 2°> and 2°< deviation from the patient’s pre-arthritic slope in the present study, that slacken and tighten the PCL in flexion, caused a loss of internal tibial orientation only at 90° and not in extension, confirming the results of in vivo and in vitro studies that PCL tension, which progressively increases with flexion, drives internal tibial rotation (Figure 4 and Figure 5) [12,13]. The PCL’s resection in the cadaveric knee reduced internal tibial rotation at high flexion angles beginning at 60° [12]. A three-dimensional fluoroscopic analysis of a deep knee bend in patients with a PCL injury in one knee and the other intact showed a decreased internal tibial rotation throughout the range of flexion in the PCL-deficient knee, which correlated with patellar tilt (R^2^ = 0.73) and medial–lateral patellar translation (R^2^ = 0.63) [13,31]. Hence, surgeons and bioengineers should consider restoring the native knee’s kinematic coupling between internal tibial rotation and patellofemoral tracking and loading when developing surgical techniques such as TKA [31].

The present study suggests that the correct target for setting the tibial component with a calipered KA TKA and PCL retention is the patient’s individual pre-arthritic slope within a tolerance of ±2° because the 15–19° range of internal tibial rotation was comparable to the native knee and more significant deviations increase the risk of tibial component failure [4,6,11,17]. The fixed 3–7° slope range recommended for MA does not account for the 20° inter-individual range of the pre-arthritic posterior slope and should not be used with a calipered KA as only 33% (7 of 21) of the tibial components in the present study fit within this range [8,9,10,17]. MA surgeons commonly use techniques such as increasing the posterior slope and PCL recession and release to increase knee flexion; however, they cause a loss of internal tibial rotation and risk tibial component failure [3,5]. A Calipered KA that sets the components patient-specific to restore the patient’s individual pre-arthritic joint lines within ±0.5 mm has the biomechanical advantage of retaining the PCL and restoring native knee tibial compartment forces and laxities during passive flexion without ligament release [23,24,25,32,33,34].

The present study has several limitations. These results are from a case series of two surgeons who require confirmation by others. The evaluation of the insert goniometer was with a medial ball-in-socket and a flat lateral insert designed to replicate the dynamic conformity of the native knee described by Freeman and Pinskerova [11,35,36,37]. It might function differently with posterior-stabilized, PCL-retaining, and ultra-congruent insert geometries that are less-conforming medially and more-constrained laterally with a posterior rim that stops internal rotation, such as a chock block. A medial concavity shallower than the ball-in-socket conformity enables the femur to translate anteriorly and posteriorly, thereby lowering the PCL’s tension in flexion which drives internal tibial rotation. The internal tibial rotation might be less for implants placed with MA since the components commonly deviate from the patient-specific native joint lines, and ligaments are released to slacken an over-tensioned TKA [38,39,40].

## 5. Conclusions

The present study measured the passive internal tibial rotation with a novel goniometric insert between full extension and 90 degrees of flexion with the tibial component set to restore and deviate 2°> and 2°< from the patient’s individual pre-arthritic slope in 21 patients treated with a calipered KA TKA and PCL retention, and showed that the pre-arthritic slope is the correct target as this resulted in a 15–19° range of internal tibial rotation, comparable to the 15–18° range reported for the native knee, whereas 2° deviations in slope caused a loss of tibial rotation.

## Figures and Tables

**Table 1:** Preoperative Patient Demographics and Clinical and Radiographic Characteristics of Included and Not-Included Patients.

Preoperative Demographics and Clinical and Radiographic Characteristics	Included Patients *N* = 21	Not-Included Patients *N* = 15	Significance
	DEMOGRAPHICS		
Age (years)	70 (±7.9)	68 (±8.8)	n.s.
Sex (male)	8 (38%)	7 (47%)	n.s.
Body Mass Index (kg/m^2^)	29.2 (±5.3)	30.2 (±4.4)	n.s.
	PREOPERATIVE MOTION, DEFORMITY, ACL CONDITION, AND KELLGREN-LAWRENCE SCORE		
Extension (degrees)	7 (±5)	7 (±8)	n.s.
Flexion (degrees)	112 (±6.4)	110 (±8.7)	n.s.
Varus (+)/Valgus (−) Deformity (degrees)	−12.2 (±3.1)	−10.8 (±3.1)	n.s.
Kellgren- Lawrence Score	3.6 (±0.6)	3.4 (±0.5)	n.s.
	PREOPERATIVE FUNCTION		
Oxford Score (48 is best, 0 is worst)	21 (±8.4)	16 (±6.5)	n.s.
Knee Society Score	38 (±11.7)	38 (±16.4)	n.s.
Knee Function Score	55 (±21.5)	46 (±16.1)	n.s.
